# *Mycobacterium tuberculosis* Phosphoribosylpyrophosphate Synthetase: Biochemical Features of a Crucial Enzyme for Mycobacterial Cell Wall Biosynthesis

**DOI:** 10.1371/journal.pone.0015494

**Published:** 2010-11-15

**Authors:** Anna P. Lucarelli, Silvia Buroni, Maria R. Pasca, Menico Rizzi, Andrea Cavagnino, Giovanna Valentini, Giovanna Riccardi, Laurent R. Chiarelli

**Affiliations:** 1 Dipartimento di Genetica e Microbiologia, Università degli Studi di Pavia, Pavia, Italy; 2 DISCAFF, Università del Piemonte Orientale “A. Avogadro”, Novara, Italy; 3 Dipartimento di Biochimica “A. Castellani”, Università degli Studi di Pavia, Pavia, Italy; University of Delhi, India

## Abstract

The selection and soaring spread of *Mycobacterium tuberculosis* multidrug-resistant (MDR-TB) and extensively drug-resistant strains (XDR-TB) is a severe public health problem. Currently, there is an urgent need for new drugs for tuberculosis treatment, with novel mechanisms of action and, moreover, the necessity to identify new drug targets. Mycobacterial phosphoribosylpyrophosphate synthetase (*Mtb*PRPPase) is a crucial enzyme involved in the biosynthesis of decaprenylphosphoryl-arabinose, an essential precursor for the mycobacterial cell wall biosynthesis. Moreover, phosphoribosylpyrophosphate, which is the product of the PRPPase catalyzed reaction, is the precursor for the biosynthesis of nucleotides and of some amino acids such as histidine and tryptophan. In this context, the elucidation of the molecular and functional features of *Mtb*PRPPase is mandatory. *Mtb*PRPPase was obtained as a recombinant form, purified to homogeneity and characterized. According to its hexameric form, substrate specificity and requirement of phosphate for activity, the enzyme proved to belong to the class I of PRPPases. Although the sulfate mimicked the phosphate, it was less effective and required higher concentrations for the enzyme activation. *Mtb*PRPPase showed hyperbolic response to ribose 5-phosphate, but sigmoidal behaviour towards Mg-ATP. The enzyme resulted to be allosterically activated by Mg^2+^ or Mn^2+^ and inhibited by Ca^2+^ and Cu^2+^ but, differently from other characterized PRPPases, it showed a better affinity for the Mn^2+^ and Cu^2+^ ions, indicating a different cation binding site geometry. Moreover, the enzyme from *M. tuberculosis* was allosterically inhibited by ADP, but less sensitive to inhibition by GDP. The characterization of *M. tuberculosis* PRPPase provides the starting point for the development of inhibitors for antitubercular drug design.

## Introduction

*Mycobacterium tuberculosis*, which is the etiologic agent of tuberculosis (TB), was discovered in 1882 by the German physician Robert Koch. TB was already then considered one of the most dangerous infectious diseases but, continues to still be, unfortunately, a major cause of death in underdeveloped nations, and a re-emerging disease in developed countries. Moreover, TB is currently endemic in the regions of sub-Saharan Africa, where susceptibility of HIV-infected people in developing the disease continuously increases [1].

According to the World Health Organization (WHO), in 2006 there were 9.2 million new cases of TB, and 1.7 million deaths from the disease, of which 95% occurred in low-income countries [2]. TB treatment is made more difficult by the emergence of multidrug resistant strains (MDR-TB), i.e. strains resistant to two of the first-line drugs, either isoniazid or rifampicin. MDR-TB demands treatment with second-line drugs [3]–[4]. Lately, a still more dangerous form of tuberculosis, i.e. extensively drug-resistant tuberculosis (XDR-TB), has been identified in all regions of the world and is becoming an alarming growing global health problem [5].

For these reasons, an emergence of a global plan to stop TB is necessary and needs the designing of new drugs and the identification of new molecular targets [6]–[7].

Recent studies have shown that, because of the mycobacterial cell wall's importance as a virulence factor in pathogenicity, it is thus rich in promising drug targets [8]. The mycobacterial cell wall structure is very complex and highly hydrophobic. It is characterized on the outer side by a mycolic acid layer and on the inner side by a peptidoglycan layer. These two layers are linked together by an arabinogalactan complex. It has been demonstrated that enzymes involved in arabinogalactan biosynthesis are essential for the livelihood of *M. tuberculosis* [9]. This makes these enzymes ideal targets for designing new antitubercular drugs.

Recently, Makarov *et al.* [10] demonstrated that benzothiazinones, which are a new generation class of antitubercular drugs, act inhibiting *M. tuberculosis* DprE1 activity, an essential membrane associated enzyme [11]–[12] that works in concert with the DprE2 enzyme in catalyzing the epimerization of decaprenylphosphoryl-ribose (DPR) to decaprenylphosphoryl-arabinose (DPA), which is a precursor for arabinan synthesis [12]. It is noteworthy that without DPA, a complete mycobacterial cell wall cannot be produced [12].

Within the DPA biosynthesis pathway, other enzymes could be considered potential antitubercular targets such as the phosphoribosylpyrophosphate synthetase (PRPPase).

**Figure 1:**
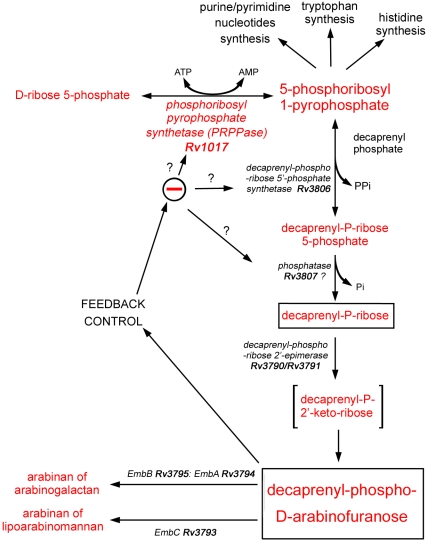
The biosynthesis pathway of decaprenylphosphoryl arabinose in mycobacteria. The figure was adapted from Wolucka BA (2008) Biosynthesis of D-arabinose in mycobacteria – a novel bacterial pathway with implications for antimycobacterial therapy. FEBS Journal 275: 2691–2711. Reproduced with permission.

PRPPase (EC 2.7.6.1) catalyzes the transfer of the β,γ-pyrophosphoryl group from the Mg^2+^ ATP complex (Mg-ATP) to ribose 5-phosphate (R5P) in order to form 5-phosphoribosyl-1-pyrophosphate (PRPP) [13], which is the precursor for the biosynthesis of purine and pyrimidine nucleotides, as well as of pyridine nucleotides coenzymes and of the amino acids histidine and tryptophan [14]. *M. tuberculosis* PRPPase (*Mtb*PRPPase), which is encoded by the *rv1017c* (*prsA*) gene, is also involved in the biosynthesis of DPA [12] (Fig. 1).

Three different classes of PRPPase have been described so far with distinctive enzymatic properties, such as the requirement of phosphate ions for activity and allosteric regulation and specificity for the diphosphoryl donor. Most PRPPases belong to class I, and are also named “classical” PRPPases. These enzymes, which require phosphate and Mg^2+^ ions, are allosterically inhibited by ADP and, possibly, by other nucleotides, and exclusively use ATP or, in some instances, also dATP as diphosphoryl donors [15]–[17]. Class II PRPPases are specific for plants and are characterized by the independence of phosphate ions and the lack of allosteric inhibition by purine ribonucleoside diphosphates. Moreover, class II PRPPases have a broad specificity for diphosphoryl donors using GTP, CTP or UTP in addition to ATP and dATP [18]–[20]. Finally, a new class III PRPPase has been recently described, from the archaeon *Methanocaldococcus jannaschii*. This enzyme is activated by phosphate and uses ATP as a diphosphoryl donor. Conversely, it is devoid of the allosteric site for ADP [21].

The crystal structures of *Bacillus subtilis* and human isoform 1 (class I) [22]–[23], as well as *M. jannaschii* (class III) PRPPase have been solved [21]. Class I enzymes are hexamers of identical subunits, which consist of two domains that are organized as a propeller with the N-terminal domains at the centre and the C-terminal domains on the outside. The substrates binding sites are located at the interface between the domains of each subunit, whereas the allosteric sites are at the interface between the three subunits of the hexamer. On the contrary, the class III PRPPase is tetrameric. The active sites are at the interface between the domains of the subunits, although no allosteric sites have been found [21].

Our laboratory is aimed at producing enzymes involved in the DPA synthesis, such as DprE1 [10], for structural studies and drug design, as we believe that the enzymes belonging to this pathway could represent a “weak ring of the chain” [24].

In this context, the PRPPase enzyme seems very promising being essential as shown by Himar1-based transposon mutagenesis in the *M. tuberculosis* H37Rv strain [25] and is furthermore involved in two important pathways: the DPA, and purine/pyrimidine nucleotides biosyntheses.

In this work, the biochemical characterization of the *M. tuberculosis* PRPPase obtained in recombinant form is reported, as a basis for the identification of a potential antitubercular drug target.

## Materials and Methods

### Strains and Growth Conditions

All cloning steps were performed in *Escherichia coli* DH5α grown in Luria-Bertani (LB) broth or on LB agar. The expression strain was *E. coli* BL21(DE3)pLysS. When necessary, antibiotics (Sigma) were added at the following concentrations: ampicillin, 100 µg/ml; chloramphenicol, 34 µg/ml; kanamycin, 50 µg/ml. All strains were grown aerobically at 37°C with shaking at 200 rpm.

### Cloning of *rv1017c* Gene in pET28-a Expression Vector

The *rv1017c* gene (*prsA*) encoding *Mtb*PRPPase, was amplified by PCR from the genomic DNA of *M. tuberculosis* H37Rv using Taq DNA Polymerase (Qiagen) with primers Rv101728aF (5′-TTGGATCCTTGAGCCACGACTGG-3′; *Bam*HI restriction site is underlined) and Rv1017R (5′-TTAAGCTTCTATGCGTCCCCGTCG-3′; *Hind*III restriction site is underlined). The PCR reaction was performed by using the MJ Mini Personal Thermal Cycler (BioRad). The resulting amplified fragment (981 bp) was purified with a Wizard PCR Prep mini-column (Promega), digested with *Bam*HI and *Hind*III restriction endonucleases, and cloned into pET28-a expression vector (Novagen) by means of T4 DNA ligase in order to form the pET28-a/*rv1017* construct which carries a fusion of six histidine residues at its N-terminus [26]. Restriction enzymes and T4 DNA ligase were purchased from GE-Healthcare and used following the manufacturer's instructions.

### *Mtb*PRPP Synthetase Heterologous Production and Purification

*E. coli* BL21(DE3)pLysS cells were electroporated with the pET28-a/*rv1017* construct and grown on LB agar plates containing kanamycin (50 µg/ml) and chloramphenicol (34 µg/ml). Roughly 100 colonies were inoculated in 2 litres of ZYP-5052 autoinducing medium [27] containing kanamycin (50 µg/ml) and chloramphenicol (34 µg/ml), and incubated at 37°C for 3 hrs and at 17°C o. n. with orbital shaking at 200 rpm. Cells were collected by centrifugation (at 6000×*g* for 10 min at 4°C), washed with cold PBS and stored at −20°C.

In order to purify the enzyme, frozen cells were suspended in 250 ml buffer A (sodium phosphate pH 8.0, 300 mM NaCl, 10 mM imidazole), supplemented with a protease inhibitor cocktail (Sigma-Aldrich), sonicated at 800 W for 6 minutes, cleared by ultracentrifugation, and the supernatant was applied to a HisTrap HP column (GE-Healthcare) equilibrated in buffer A. Proteins were eluted with scalar concentration (20 to 500 mM) of imidazole in buffer A and fractions containing *Mtb*PRPPase activity were collected, concentrated and applied to a HiLoad 16/60 Superdex-200 column (GE-Healthcare) equilibrated in buffer B (50 mM potassium phosphate pH 8.0, 100 mM KCl). The enzyme was eluted by buffer B and fractions containing *Mtb*PRPPase activity were checked by 12% SDS-PAGE and pooled. Protein concentration was determined according to Lowry *et al.* [28].

### Molecular Mass Determination

To determine the molecular mass of the native enzyme, the purified *Mtb*PRPPase (100 µl, 0.1 mg/ml) was subjected to an analytical gel filtration on a Superose 6 HR 10/30 prepacked column (GE-Healthcare) equilibrated in buffer B. For column calibration the following proteins were used: thyroglobulin (669 kDa), ferritin (440 kDa), catalase (240 kDa), aldolase (158 kDa), albumin (68 kDa), and ribonuclease (13.7 kDa).

### Enzyme Activity Assay

*Mtb*PRPPase activity was assayed with a HPLC-based method developed in our laroratory (unpublished data), and following the AMP rate formation. The standard reaction mixture contained 50 mM potassium phosphate pH 8.0, 100 mM KCl, 2 mM Mg-ATP, 2 mM R5P, in a final volume of 100 µl. After incubation at 37°C, the reaction was stopped by adding 10% (w/v) ice-cold trichloroacetic acid, and neutralized with 200 mM K_2_CO_3_. After centrifugation, samples (10 µl) were loaded onto a Supelcosil LC-18 column (250×4.6 mm, 5 µm particle size, Supelco Analytical). Isocratic separation was performed in 20 mM potassium phosphate pH 8.0 at a flow rate of 0.8 ml/min. Analytes were monitored at 254 nm.

The nmoles of AMP produced were determined using a calibration curve obtained by injecting scalar amounts (0.06 to 20 nmol) of AMP, treated in the same way as that adopted for the enzyme assay. One unit is defined as the amount of enzyme catalyzing the production of 1 µmol of AMP per minute under conditions here described.

### Kinetic Analyses

Unless otherwise indicated, enzymatic activity was assayed at 37°C by using various concentrations of R5P and Mg-ATP under conditions identical to those described above except for substrates and effectors. The kinetic parameters were determined for R5P at 10 mM Mg-ATP and for Mg-ATP at 2 mM R5P. In all cases the reaction was initiated by adding R5P, and the enzyme activity was assayed at least with 12 different concentrations of substrate. All measurements were performed at least in triplicate. The plot of Lineweaver-Burk was used to determine V_max_ and apparent K_m_ values. The Hill plot obtained by the Enzyme Kinetic Module 1.1 (SPSS Science Software) was used to determine the apparent S_0.5_ and n_H_ values.

For the assessment of the activation by phosphate or sulfate ions, the enzyme stored in buffer B was diluted in 50 mM Tris HCl pH 8.0 buffer, containing 2mM Mg-ATP, lowering the phosphate concentration to 0.25 mM. The enzyme activity was then immediately assayed at saturating concentrations of substrates, and using as assay buffer 50 mM Tris-HCl pH 8.0, 100 mM KCl, in the presence of different concentrations of potassium phosphate or ammonium sulfate.

### Thermal Stability Assays

Thermal stability was measured by incubating the enzyme (100 µg/ml) at given temperatures in buffer B, in the absence and in the presence of ligands. Samples were removed at intervals and immediately assayed as described above.

Relative activity was expressed as percentage of the enzyme activity before the incubation. t_1/2_ is the time required by the enzyme to lose 50% of its initial activity at a given temperature.

The thermal denaturation was also measured by circular dichroism spectropolarimetry. Thermal unfolding was followed by continuous measurements of ellipticity at 220 nm at the temperature range 50–90°C under a constant heating rate of 1°C/min, and with a Jasco J-710 spectropolarimeter (Jasco Europe, Cremella, Italy) equipped with a Neslab RT-11 programmable water bath (Thermo Fisher Scientific, Waltham, MA, USA) and a 1 mm path-length cuvette. Protein concentration was 0.1 mg/ml in buffer B. The midpoint temperatures (T_m_) were calculated from curves fitting.

### Homology Modelling of *Mtb*PRPPase

The three dimensional structure of *Mtb*PRPPase was modelled using, as the template, the atomic coordinates of the X-ray crystal structure of the human ortholog in complex with AMP, cadmium and sulfate ion (PDB code 2HCR) [23]. The program SWISS-PDBviewer in conjunction with the SWISS-MODEL server (http://www.expasy.org/spdbv/) was employed for building and optimizing the model. The stereochemistry of the predicted structure has been assessed with the program PROCHECK [29]. 92.0% of residues felt in the most favoured region of the Ramachandran plot, 8.0% in the additional allowed region with no detected outliers. The crystal structure of human PRPPase and the modelled *Mtb*PRPPase structure can be superimposed with a r.m.s.d. of 0.5 Å based on 303 Ca pairs (the two enzymes share a sequence identity of 44%). The model of the *Mtb*PRPPase-AMP complex was obtained by superposing the predicted *M. tuberculosis* structure onto the crystal structure of human template and pasting the AMP molecule into the *M. tuberculosis* modelled structure. Figures were generated with the program Pymol [30].

## Results

### Heterologous Expression and Purification of *M. tuberculosis* PRPPase

The recombinant *Mtb*PRPPase was expressed in *E. coli* BL21(DE3)pLysS cells, and purified to homogeneity as described in the “Material and Methods” section. The typical yield was about 20 mg of purified *Mtb*PRPPase from 1 litre of culture. The specific activity, under standard conditions, was 59.7 U/mg. No detectable activity was found with Mg-GTP used as substrate. As phosphate (P_i_) has been reported to be indispensable in preserving protein stability of PRPPases, the *Mtb*PRPPase was maintained in 50 mM phosphate, pH 8.0 [16]–[17], [23]. In actual fact, dialysis against buffers such as 50 mM Tris-HCl, pH 8.0 or 50 mM Hepes-NaOH, pH 8.0 resulted in a protein precipitation and complete loss of activity. The addition of 50 mM ammonium sulfate or 5 mM Mg-ATP to Tris-HCl, pH 8.0 allowed the enzyme to preserve 20% of initial activity after a period of 16 hours, whereas full activity was maintained with the addition of 50 mM P_i_.

### Main Characteristics of *Mtb*PRPPase

**Figure 2:**
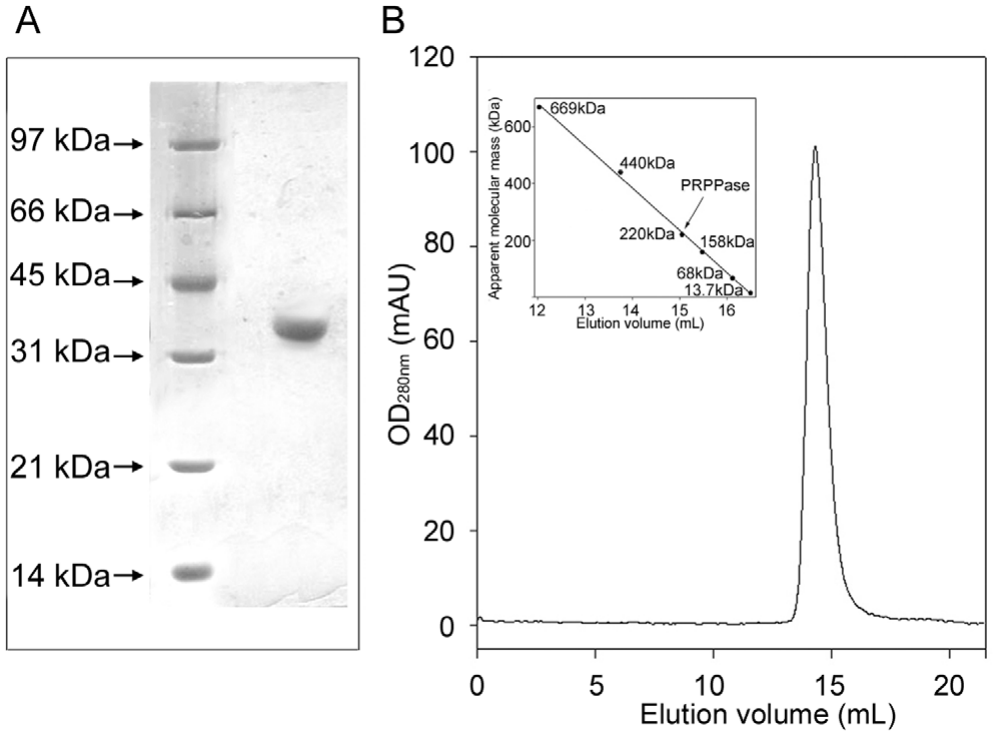
Assessment of the oligomeric state of *Mtb*PRPPase. (A) SDS-PAGE of the purified *Mtb*PRPPase. The enzyme was run in parallel with molecular mass standards on a 12% gel and stained with Coomassie Blue R-250. Molecular mass markers were, from the top, 97, 66, 45, 31, 21.3 and 14.4 kDa, respectively. (B) Elution profile of *Mtb*PRPPase from a Superose 6 column. The enzyme was subjected to an analytical gel-filtration on a Superose 6HR 10/30 prepacked column. The position of the peak corresponds to a protein of 220 kDa. The inset shows the calibration curve, prepared as reported in “Materials and Methods”.

*Oligomeric state*—The enzyme migrated in 12% SDS-PAGE as a protein of apparent molecular mass of approximately 35 kDa (Fig. 2A) and eluted from a Superose 6 column as a single simmetric peak, corresponding to a 220 kDa protein (Fig. 2B). These results indicated that the recombinant *Mtb*PRPPase was a hexamer of identical subunits.

**Figure 3:**
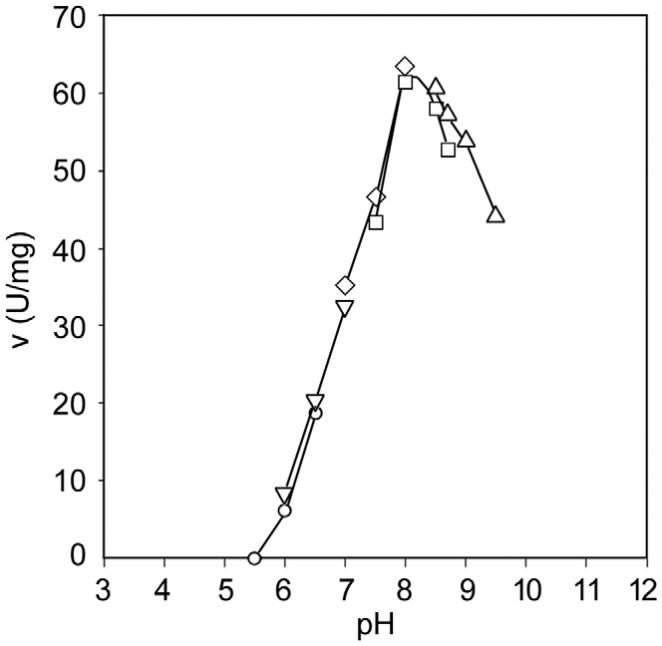
pH-activity profile of *Mtb*PRPPase. The effect of pH on the activity of *Mtb*PRPPase was determined at 2 mM R5P and 5 mM Mg-ATP, using the following buffers (100 mM): MES (○, pH range 5.5–6.5); PIPES (▿, 6–7); TES (⋄, 7–8), EPPS (□, 7.5–8.4); and sodium bicarbonate (▵, 8.25–9.5). All buffers contained 50 mM P_i_.

*Dependence on pH*—The pH-activity profile for *Mtb*PRPP is shown in Figure 3. The enzyme exhibited preference for high pH values, showing an optimum at a pH value close to 8, and possessing nearly 70% of its maximal activity at pH 9.5. The activity at pH 7 was only 57% of the maximal one. The pH profile exhibited by *Mtb*PRPPase approached that of *B. subtilis* enzyme [31]

**Figure 4:**
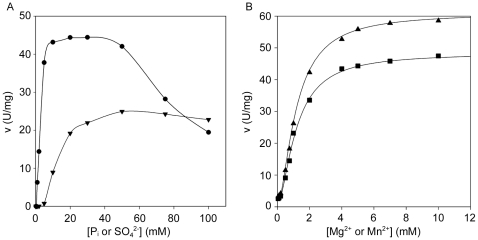
Activation of *Mtb*PRPPase by ions. (A) *Mtb*PRPPase activity response to different concentrations of phosphate (•) and sulfate (▾) anions. Concentrations of R5P and Mg-ATP were fixed at 2 mM and 5 mM, respectively. Enzyme assays were performed in 50 mM Tris-HCl pH 8.0 as reported in the “Material and Methods” section. (B) *Mtb*PRPPase activation by different concentrations of Mg^2+^ (▪) and Mn^2+^ (▴) cations. Enzyme assays were performed at 2 mM R5P and 0.5 mM Mg-ATP fixed concentrations.

*Requirements for inorganic phosphate*—PRPPases are known to require phosphate for their activity [16]–[17], [23]. *Mtb*PRPPase resulted to be actually dependent on P_i_ for its activity: the optimal P_i_ concentration ranged from 10 mM to 40 mM; higher concentrations of P_i_ were inhibitory (50% inhibition at 100 mM P_i_) (Fig. 4A). SO_4_^2−^ ions were also able to stimulate the enzyme activity, but with respect to P_i_, were less effective and required higher concentrations (40–60 mM) in order to exhibit maximal activation (Fig. 4A). On the contrary, SO_4_^2−^, at concentrations up to 100 mM, were only faintly inhibitory.

*Activation by divalent cations*—It has been reported that PRPPases are activated by free divalent cations. At subsaturating Mg-ATP concentrations, *Mtb*PRPPase reached half-maximum activation at approximately 1 mM free ions (Mg^2+^ and Mn^2+^, 1.2 mM and 1.1 mM, respectively), although the maximal activity reached in the presence of 5 mM Mg^2+^ resulted to be roughly 80% of that in the presence of 5 mM Mn^2+^ (Fig. 4B)

### Steady State Kinetics as a Function of Substrates Concentration

**Figure 5:**
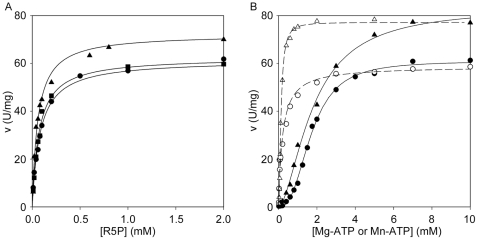
Steady state kinetics of *Mtb*PRPPase. (A) Steady state kinetics of *Mtb*PRPPase as a function of R5P. All assays were performed at fixed 10 mM Mg-ATP, in the absence of free divalent ions (•), in the presence of 5 mM MgCl_2_ (▪), and in the presence of 5 mM MnCl_2_ (▴). (B) Steady state kinetics of *Mtb*PRPPase as a function of Mg-ATP. All experiments were performed at fixed 2 mM R5P, in the absence (**•**) and in the presence (○) of 5 mM MgCl_2_, and as a function of Mn-ATP in the absence (▴) and in the presence (▵) of 5 mM MnCl_2_. Enzyme assay conditions are reported in the “Material and Methods” section.

Steady state kinetics of the recombinant *Mtb*PRPPase as a function of R5P and Mg-ATP, are shown in Figure 5. Main kinetic parameters are summarized in Tables 1 and 2.

**Table 1:** Main kinetics parameters of *Mtb*PRPPase towards R5P in the absence and in the presence of free divalent cations.

	k_cat_ (s^−1^)	K_m_ (mM)	k_cat_/K_m_ (s^−1^ mM^−1^)
No addition	37.0±1.8	0.071±0.006	521.1
+Mg^2+^	35.1±2.3	0.070±0.015	501.4
+Mn^2+^	44.7±2.6	0.060±0.008	745.0

When present, free cations were at 5 mM fixed concentration.

**Table 2:** Main kinetics parameters of *Mtb*PRPPase towards Mg-ATP and Mn-ATP in the absence and in the presence of free divalent cations.

	k_cat_ (s^−1^)	S_0.5_ (mM)	n_H_	k_cat_/S_0.5_ (s^−1^ mM^−1^)
Mg-ATP	35.5±2.3	1.71±0.09	2.6±0.3	20.8
Mn-ATP	46.3±2.4	1.78±0.11	1.9±0.2	26.0
Mg-ATP+Mg^2+^	34.6±3.0	0.26±0.05	1.0±0.2	133.1
Mn-ATP+Mn^2+^	45.1±2.4	0.11±0.01	1.0±0.1	410.0
Mg-ATP+Mn^2+^	44.3±2.4	0.11±0.01	1.0±0.1	402.7

When present, free cations were at 5 mM fixed concentration.

At saturating concentration of Mg-ATP, the enzyme exhibited hyperbolic response to R5P (Fig. 5A), with an apparent K_m_ of 0.071 mM. On the contrary, at saturating R5P concentration, it showed sigmoidal behaviour towards Mg-ATP (Fig. 5B), with an apparent S_0.5_ of 1.71 mM and a Hill coefficient (n_H_) of 2.6.

The presence of 5 mM free Mg^2+^ in kinetics towards R5P did not alter the curve profile, whereas 5 mM Mn^2+^ raised the maximal activity to 120% (Fig. 5A). As for the response of the enzyme towards Mg-ATP, the presence of 5 mM free Mg^2+^ converted the sigmoid curve into a hyperbole, lowering the apparent S_0.5_ value and leaving the V_max_ value unchanged (Fig. 5B and Table 2). A similar effect was obtained by the presence of 5 mM Mn^2+^ to the kinetics *versus* Mn-ATP (Fig. 5B and Table 2). Notably, the presence of 5 mM Mn^2+^ in the kinetics *versus* Mg-ATP (curve profile not shown) led to kinetic parameters which were nearly identical to those obtained for the kinetics towards Mn-ATP (Table 2).

### Inhibition by Divalent Cations

**Figure 6:**
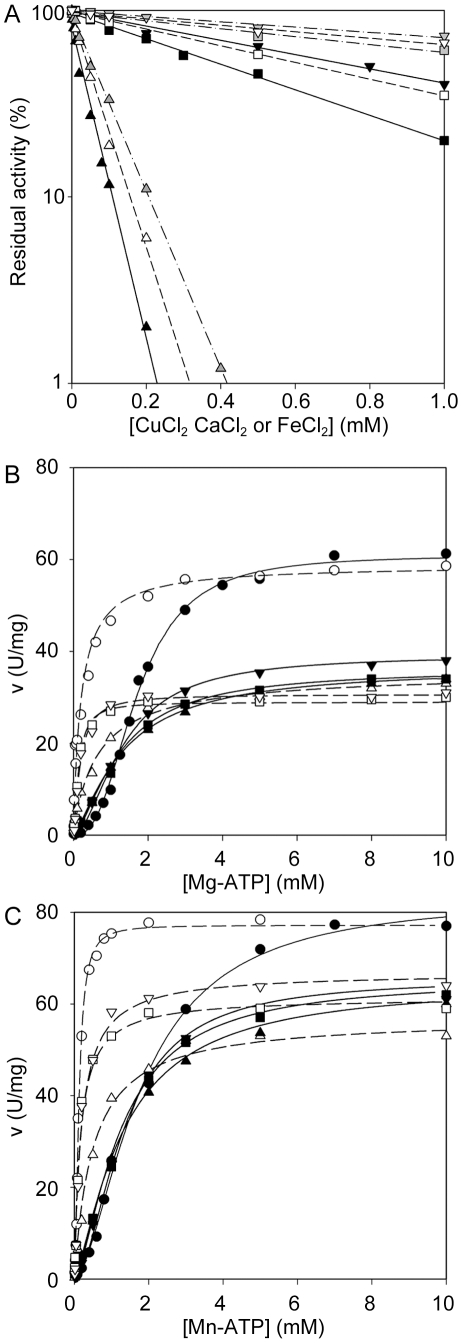
Inhibition of *Mtb*PRPPase by divalent cations. (A) Response of PRPPase activity to CuCl_2_ (▴), CaCl_2_ (▾) and FeCl_2_ (▪) different concentrations. All measurements were performed at 2 mM R5P and 5 mM Mg-ATP, in the absence (black symbols), and in the presence of 5 mM MgCl_2_ (white symbols) or 5 mM MnCl_2_ (gray symbols). (B) Steady state kinetics *vs* Mg-ATP, at 2 mM R5P in the absence (**•**) and in the presence of 0.02 mM CuCl_2_ (▴), 0.8 mM CaCl_2_ (▾) and 0.4 mM FeCl_2_ (▪), concentrations. Measurements were performed either in the absence (filled symbols) or in the presence (open symbols) of 5 mM MgCl_2_ (C) Steady state kinetics *vs* Mn-ATP, at 2 mM R5P in the absence (**•**) and in the presence of 0.02 mM CuCl_2_ (▴), 0.8 mM CaCl_2_ (▾) and 0.4 mM FeCl_2_ (▪), concentrations. Measurements were performed either in the absence (filled symbols) or in the presence (open symbols) of 5 mM MnCl_2_.

Divalent cations, such as Ca^2+^ or Cd^2+^, are reported to inhibit PRPPases [31]. Figure 6A reports the inhibition curves of CuCl_2_, CaCl_2_ and FeCl_2_ at 5mM Mg-ATP. All ions resulted to be inhibitory, Cu^2+^ being the most effective, with an IC_50_ (inhibitor concentration lowering enzyme activity to 50%) value of 0.02 *versus* 0.4 and 0.8 mM of Fe^2+^ and Ca^2+^, respectively. The presence of Cu^2+^, Ca^2+^or Fe^2+^ at a concentration equal to their IC_50_ left the affinity for Mg-ATP unchanged or even slightly increased, as shown by the kinetics towards this substrate (Fig. 6B, Tables 3 and 4). In addition, these ions reduced, but did not completely abolish, the cooperativity towards Mg-ATP (n_H_ value reduced up to 1.4 in the case of Cu^2+^, Table 3). The inhibition was not even removed by using fully activating concentrations of free MgCl_2_, although in the presence of Mg^2+^ the curves *vs* Mg-ATP became hyperbolic. V_max_ values remained similar to those obtained in the presence of inhibitory ions alone (Fig. 6B, Table 3). Comparable inhibitory effects were also observed when Mn-ATP was used as the variable substrate, although the V_max_ values were slightly reduced. The addition of free Mn^2+^ abolished the enzyme cooperativity towards the nucleoside triphosphate, leaving the V_max_ values almost unchanged (Fig 6C, Table 4).

**Table 3:** Kinetics parameters of *Mtb*PRPPase *vs* Mg-ATP with different inhibitors in the absence and in the presence of 5mM MgCl_2_.

	no addition				+MgCl_2_			
	k_cat_ (s^−1^)	S_0.5_ (mM)	n_H_	k_cat_/S_0.5_ (s^−1^ mM^−1^)	k_cat_ (s^−1^)	S_0.5_ (mM)	n_H_	k_cat_/S_0.5_ (s^−1^ mM^−1^)
No addition	35.5±2.3	1.71±0.09	2.6±0.3	20.8	34.6±3.0	0.26±0.05	1.0±0.2	133.1
CuCl_2_ 0.02 mM	21.2±1.7	1.32±0.21	1.4±0.2	16.1	20.6±1.3	0.67±0.05	1.0±0.1	30.7
CaCl_2_ 0.80 mM	22.8±1.9	1.33±0.11	1.8±0.3	17.1	19.8±0.9	0.18±0.02	1.2±0.2	110.0
FeCl_2_ 0.40 mM	20.9±1.8	1.25±0.21	1.5±0.2	16.7	18.8±0.8	0.14±0.01	1.1±0.2	134.3

**Table 4:** Kinetics parameters of *Mtb*PRPPase *vs* Mn-ATP with different inhibitors in the absence and in the presence of 5mM MnCl_2_.

	no addition				+MnCl_2_			
	k_cat_ (s^−1^)	S_0.5_ (mM)	n_H_	k_cat_/S_0.5_ (s^−1^ mM^−1^)	k_cat_ (s^−1^)	S_0.5_ (mM)	n_H_	k_cat_/S_0.5_ (s^−1^ mM^−1^)
No addition	46.3±2.4	1.78±0.11	1.9±0.2	26.0	45.1±2.4	0.11±0.01	1.0±0.1	410.0
CuCl_2_ 0.02 mM	37.1±2.0	1.38±0.18	1.3±0.2	26.8	32.6±1.4	0.50±0.06	1.0±0.1	65.2
CaCl_2_ 0.80 mM	38.6±1.6	1.35±0.11	1.5±0.1	28.5	32.2±1.6	0.20±0.03	1.1±0.2	161.0
FeCl_2_ 0.40 mM	39.5±3.2	1.43±0.23	1.4±0.2	27.6	35.1±1.5	0.16±0.02	1.1±0.3	219.4

### Inhibition by ADP

**Figure 7:**
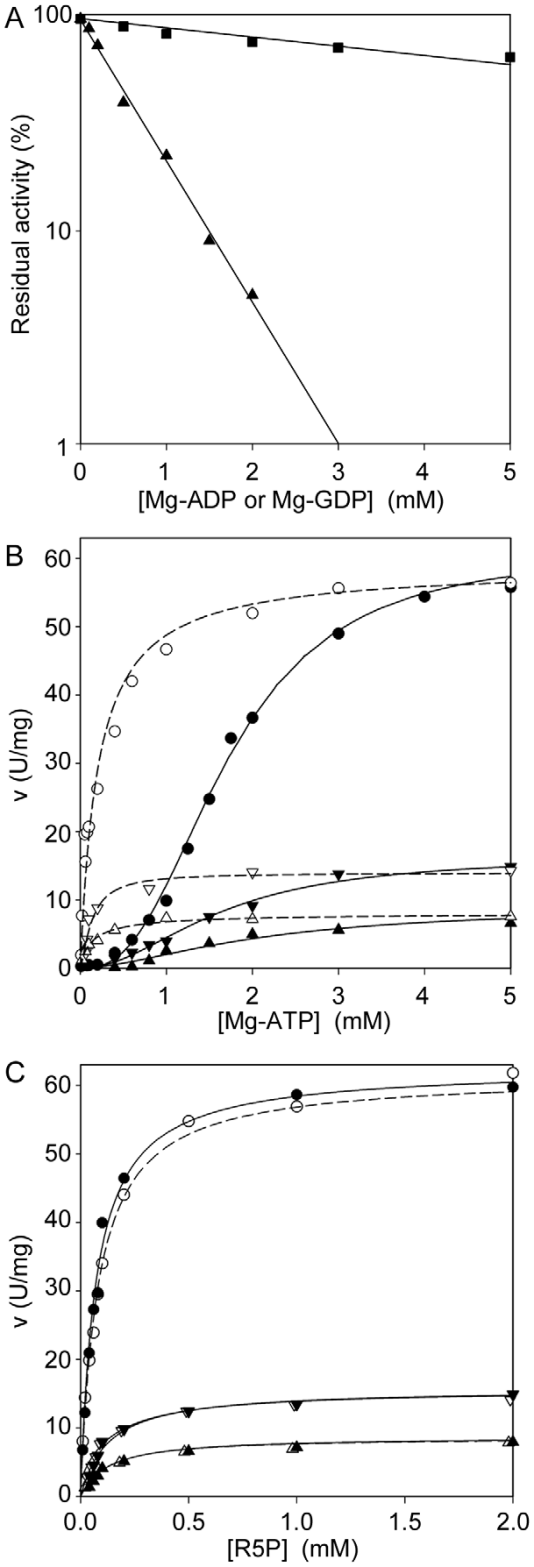
Inhibition of *Mtb*PRPPase by nucleoside diphosphates. (A) Response of *Mtb*PRPPase activity to Mg-ADP (▴), and Mg-GDP (▪) different concentrations. All measurements were performed at 2 mM R5P and 1 mM Mg-ATP. (B) Steady state kinetics *vs* Mg-ATP, at 2 mM R5P, in the presence of 1 mM Mg-ADP (▴) and 0.5 mM Mg-ADP (▾), in the absence (filled symbols) or in the presence (open symbols) of 5 mM MgCl_2_. (C) Steady state kinetics *vs* R5P, at 5 mM Mg-ATP, in the presence of 1 mM Mg-ADP (▴) and 0.5 mM Mg-ADP (▾), in the absence (filled symbols) or in the presence (open symbols) of 5 mM MgCl_2_. The circles indicate the kinetics in the absence of the inhibitor. Notably, all measurements were performed in 50 mM potassium phosphate buffer, pH 8.0.

Class I PRPPases are reported to be allosterically inhibited by ADP or by GDP [17]. The inhibition curves of Mg-ADP and Mg-GDP at subsaturating concentrations of Mg-ATP and in the presence of 50mM P_i_ (Fig. 7A) showed that *Mtb*PRPPase was weakly sensitive to GDP (IC_50_>5 mM), whereas it was highly inhibited by ADP (IC_50_ 0.4 mM). The degree of inhibition by ADP was higher at lower concentration of P_i_ (IC_50_, 0.26 mM at 5 mM P_i_, Fig. S1), suggesting that ADP inhibition hindered P_i_ in its activatory ability. Thus, inhibition by ADP and activation by P_i_ resulted to occur by competition for binding to the same site.

To prove that ADP was actually an allosteric inhibitor of *Mtb*PRPPase, we assayed the enzyme activity at varying Mg-ATP concentration, in the presence of either 0.5 mM or 1 mM Mg-ADP, with and without 5 mM MgCl_2_ (Fig 7B). The presence of the nucleoside diphosphate lowered the V_max_ of the enzyme, without affecting both the apparent S_0.5_ and the n_H_ values. The inhibition by Mg-ADP was not removed by the presence of the activating cation (V_max_ values unchanged), although the response towards Mg-ATP became hyperbolic with an affinity for the substrate similar to that displayed in the presence of Mg^2+^ without Mg-ADP (Fig. 7B, Table 5). As for the kinetics towards R5P, the presence of Mg-ADP gave effects similar to those observed when the Mg-ATP was used as the variable substrate (Fig 7C), the V_max_ being the only kinetic parameter affected (Table 6).

**Table 5:** Kinetics parameters of *Mtb*PRPPase *vs* Mg-ATP with different ADP concentrations in the absence and in the presence of 5mM MgCl_2_.

	no addition				+MgCl_2_			
	k_cat_ (s^−1^)	S_0.5_ (mM)	n_H_	k_cat_/S_0.5_ (s^−1^ mM^−1^)	k_cat_ (s^−1^)	S_0.5_ (mM)	n_H_	k_cat_/S_0.5_ (s^−1^ mM^−1^)
No addition	35.5±2.3	1.71±0.09	2.6±0.3	20.8	34.6±3.0	0.26±0.05	1.0±0.2	133.1
Mg-ADP 0.5 mM	9.5±0.8	1.69±0.13	2.4±0.2	5.6	10.6±0.9	0.29±0.06	1.0±0.2	36.6
Mg-ADP 1.0 mM	5.0±0.7	2.10±0.31	2.5±0.3	2.4	5.3±0.4	0.31±0.02	1.0±0.1	17.1

**Table 6:** Kinetics parameters of *Mtb*PRPPase *vs* R5P with different ADP concentrations in the absence and in the presence of 5mM MgCl_2_.

	no addition			+MgCl_2_		
	k_cat_ (s^−1^)	K_m_ (mM)	k_cat_/K_m_ (s^−1^ mM^−1^)	k_cat_ (s^−1^)	K_m_ (mM)	k_cat_/K_m_ (s^−1^ mM^−1^)
No addition	37.0±1.8	0.071±0.006	521.1	35.1±2.3	0.070±0.015	501.4
Mg-ADP 0.5 mM	9.0±0.2	0.102±0.02	90.0	8.9±0.4	0.128±0.01	69.5
Mg-ADP 1.0 mM	5.1±0.1	0.121±0.01	42.5	4.9±0.11	0.121±0.02	40.5

As far as other potential inhibitors are concerned [31], it is worth mentioning that no inhibitory effects were shown by the presence of pyrimidine nucleoside mono- or diphosphates or of histidine, up to 2 mM (data not shown).

### Thermal Stability

The enzyme thermal stability was assessed either by measuring the activity at intervals after incubation at 62°C, or by monitoring the thermal unfolding at increasing temperature with circular dichroism spectropolarimetry.

**Figure 8:**
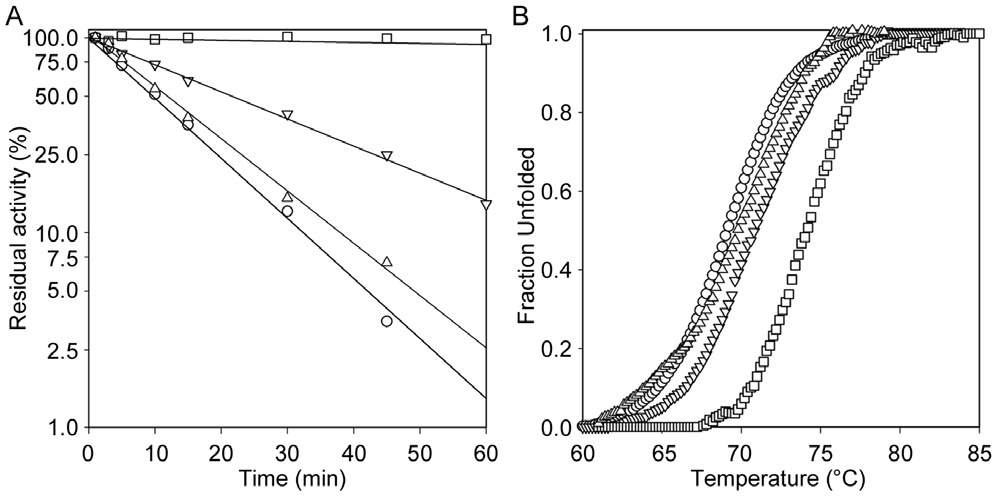
Thermal stability of *Mtb*PRPPase. (A) Thermal stability of the *Mtb*PRPPase at 62°C. The enzyme was incubated in 50 mM potassium phosphate pH 8.0, 100 mM KCl, in the absence of ligands (○) and in the presence of 5 mM Mg-ATP (□), 5 mM R5P (▿) and 5 mM MgCl_2_ (▵). Aliquots were collected at intervals for measuring residual activity. (B) Thermal unfolding kinetics of the *Mtb*PRPPase. The enzyme denaturation was monitored by circular dichroism spectropolarimetry, in the absence of ligands (○) and in the presence of 5 mM Mg-ATP (□), 5 mM R5P (▿) and 5 mM MgCl_2_ (▵).

*Mtb*PRPPase resulted to be a highly stable enzyme, losing 50% of its activity in 10 minutes of incubation at 62°C, and showing a T_m_ of 69.3°C (Table 7). Mg-ATP greatly increased the protein stability, allowing the enzyme to preserve full activity for more than one hour when incubated in the presence of this substrate. A protective effect was also exerted by R5P, although to a lesser extent (t_1/2_ 22 minutes), whereas no protection was observed in the presence of Mg^2+^ ion (Fig. 8A). Similarly, the midpoint temperatures were shifted by the presence of substrate (70.8 and 74.5°C for ATP and R5P, respectively), but not by MgCl_2_ (Fig. 8B).

**Table 7:** Thermal stability parameters of *Mtb*PRPPase in the absence and in the presence of ligands.

	t_1/2_ 62°C (min)	T_m_ (°C)
No addition	10′25″	69.3±0.1
MgCl_2_ 5 mM	11′40″	69.8±0.1
R5P 5 mM	22′25″	70.8±0.3
Mg-ATP 5 mM	Stable	74.5±0.2

### *Mtb*PRPPase Three Dimensional Structure Prediction

**Figure 9:**
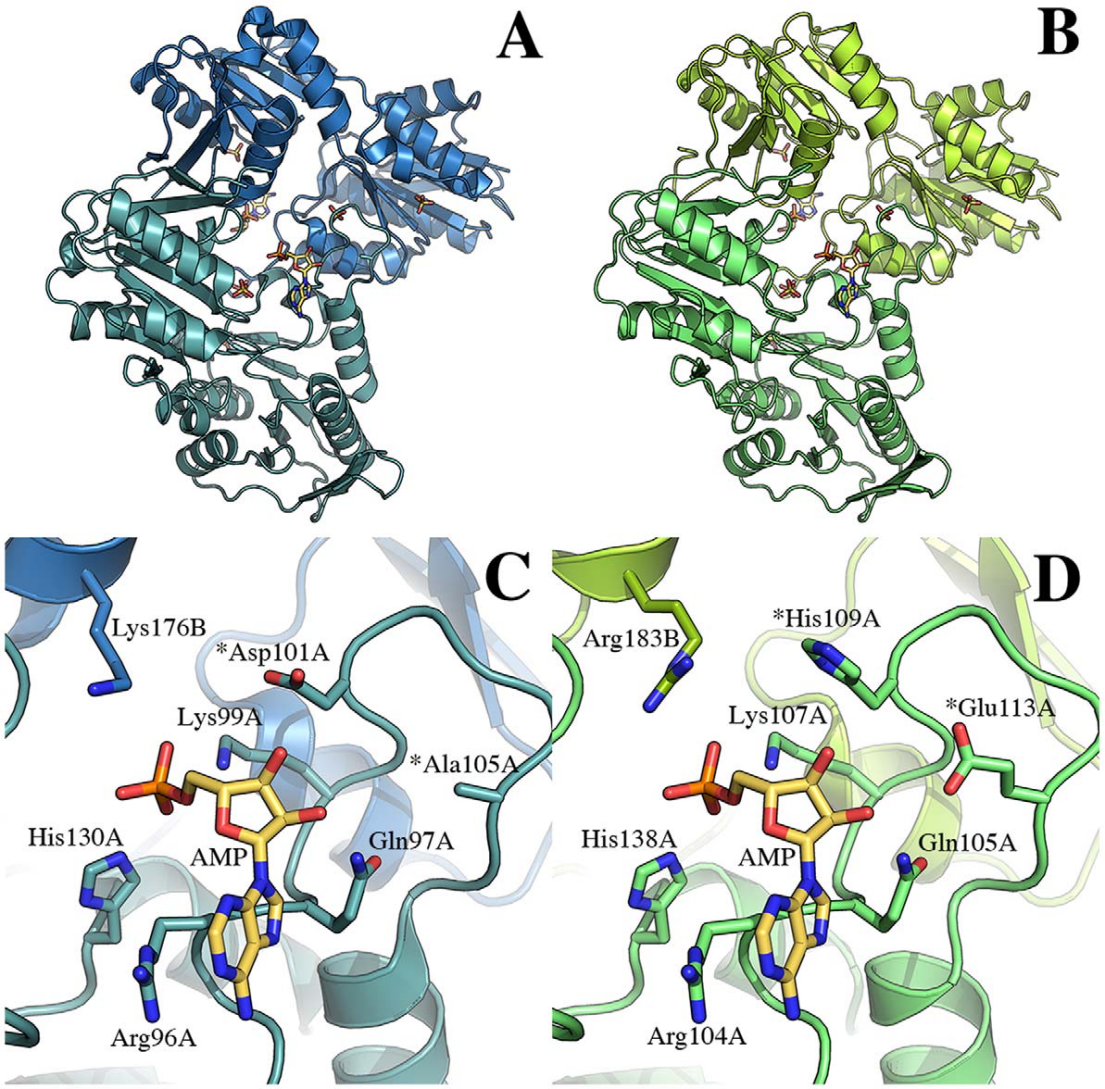
Homology modelling of *Mtb*PRPPase. (A) Ribbon representation of the crystal structure of the dimer of human PRPPase in complex with AMP, cadmium and sulfate [23] (PDB code 2HCR) that was used as the template for the homology modelling. The AMP and sulfate molecules are represented as ball-and-sticks. (B) Ribbon representation of the predicted structure of the *Mtb*PRPPase dimer. The AMP and sulfate molecules are drawn as ball-and-sticks. (C) The adenosine triphosphate binding site in human PRPPase. The AMP molecule and key residues that define the nucleotide binding pocket are labelled and represented as ball-and-sticks. (D). The adenosine triphosphate binding site as emerging in the predicted structure of *Mtb*PRPPase where the AMP and protein residues building the binding pocket are labelled and depicted as ball-and-sticks. In both (C) and (D), the asterisks indicate the two residues that are structurally not conserved in the two enzymes.

We are acutely aware of the issue of selectivity of drug action for inhibitors targeting the *Mtb*PRPPase, as the mycobacterial enzyme shares a significant degree of sequence identity with human counterpart (sequence identity of 44%). Although the identification of possible peculiar structural features to be exploited for the design of specific inhibitors must wait for the determination of the X ray crystal structure of the *Mtb*PRPPase, we carried out a prediction of its structure based on homology modelling. As expected, the overall structural organization of the mycobacterial and human enzymes appeared to be strongly conserved (Fig. 9A and 9B) as demonstrated by the observation that the two structures can be optimally superimposed with a r.m.s.d. of only 0.5 Å based on 303 Ca pairs. However, the analysis of the ATP binding pocket revealed interesting differences between the two enzymes (Fig. 9C and 9D). In particular, two major substitutions in the residues that define the nucleoside triphosphate binding site can be identified. In the *Mtb*PRPPase a glutamic acid (Glu113) occupies the structurally equivalent position of Ala105 in the human enzyme; moreover a histidine residue (His109) replaces Asp101 in the human PRPP synthetase. Since *Mtb*PRPPase shows a strong cooperativity for ATP binding, we cannot quantify the impact of these substitutions based on our predicted structure.

## Discussion

The biosynthesis pathway of decaprenylphosphoryl-arabinose has been proved to be an optimal target for antitubercular drugs [10], [12]. In this context, the characterization of *M. tuberculosis* phosphoribosylpyrophosphate synthetase, which is the enzyme catalysing the second step of this metabolic pathway, is reported. Noticeably, PRPP, which is the product of the PRPPase catalysed reaction, is also a key metabolite for the nucleotides and for the amino acids histidine and tryptophan synthesis. The *rv1017c* gene, which encodes PRPPase, is thus essential for *M. tuberculosis* growth [25].

*Mtb*PRPPase was expressed as recombinant form, purified to homogeneity and biochemically characterized. Although the biochemical characterization of the *Mtb*PRPPase was performed using the enzyme with a hexahistidine tag attached to its N-terminus, as shown in Figure S2, the tag did not affect the main kinetic properties (see Materials and Methods S1).

The enzyme exhibited a hexameric quaternary structure, specificity for Mg-ATP as substrate and requirement of phosphate for its activity. These features allowed us to label *Mtb*PRPP as class I enzyme. SO_4_^2−^ mimicked the activation by P_i_, although to a lower extent (56%). On the other hand, the inhibitory effect exhibited by P_i_ at high concentrations was negligible in the case of SO_4_^2−^. In this respect, *Mtb*PRPP turned out to be quite similar to the enzyme from *B. subtilis* and mammals [22], [32]–[33].

PRPPAses require both free Mg^2+^ ion as an essential activator and Mg-ATP as a substrate. Free ion may induce and properly stabilize the open conformation of the so-called flexible loop which binds Mg-ATP at the active site [34]–[35]. In the absence of free Mg^2+^, *Mtb*PRPPase showed homotropic cooperativity towards Mg-ATP, which was the cause of a relatively low affinity for this substrate (apparent S_0.5_, 1.71 mM). The presence of free Mg^2+^ abolished the cooperativity *versus* Mg-ATP (n_H_, 1) and lowered the apparent S_0.5_, suggesting that it activated the enzyme, behaving as an allosteric effector. Moreover, the kinetic properties displayed by *Mtb*PRPPase in the absence and in the presence of the activator Mg^2+^ could fulfil the requirements of the K-type allosteric enzyme of the model described by Monod [36]. Comparable heterotropic activation was also exerted by Mn^2+^, which resulted even more effective than Mg^2+^ (Table 2) whether the enzyme used Mg-ATP or Mn-ATP as a variable substrate. In this respect, *Mtb*PRPP showed to be different from other class I enzymes, which display maximal activation in the presence of free Mg^2+^ ions [17], [31], [37].

Thermal stability assays allowed us to evidence conformational changes caused by the presence of ligands (Fig. 8). Whereas *Mtb*PRPPase exhibited a more stable conformation in the presence of Mg-ATP (t_1/2_, >2hrs *versus* 10′20″ of the enzyme in the absence of ligands), the presence of free Mg^2+^ ions did not lead to any increased protein stability (t_1/2_, 11′40″), suggesting that the binding of the free activating ion did not induce large rearrangements of the protein. Thus, keeping in consideration previous data obtained from crystallographic studies on *B. subtilis* enzyme [22], [35], we hypothesize that the binding of the free Mg^2+^ to its site would induce a local conformational change at the active site of the single subunits, stabilizing the open conformation of the flexible loop and abolishing the cooperativity of the Mg-ATP binding sites, but leaving the overall conformation of the enzyme unchanged. On the other hand, the binding of Mg-ATP to one subunit would lead to overall enzyme conformational changes, thus inducing the stabilization of the open active site conformation in the next subunits, and increasing their affinity for Mg-ATP.

**Figure 10:**
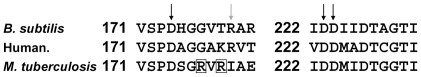
Alignment of free ion binding site sequences. Alignment of free ion binding site sequences of *B. subtilis*, human PRS1 and *M. tuberculosis* PRPPases was performed with Multalin 5.4.1. Black arrows point to the aspartic residues involved in the binding of the free ion, grey arrow to the Arg^180^ (*B. subtilis* numbering). The boxes highlight the positions of the two arginines in the *Mtb*PRPPase sequence.

Divalent cations, such as Ca^2+^ and Cd^2+^, have been reported to inhibit PRPPase activity [32], [34]. *Mtb*PRPPase was inhibited by Ca^2+^ (IC_50_, 0.8 mM), but the effect of this ion resulted to be less effective than that observed in *B. subtilis* and human enzymes [32], [34]. In actual fact, a higher inhibition was found when the enzyme activity was assayed in the presence of Cu^2+^ ions (IC_50_, 0.02 mM). However, in all cases, the reduction of the activity was accompanied by a decrease in the cooperativity towards Mg-ATP and a slight increase in the affinity for this substrate (Table 3). The inhibition was only partially removed by the addition of either free Mg^2+^ or free Mn^2+^ (V_max_ almost unchanged, but cooperativity totally abolished). In addition, in the case of Cu^2+^, the presence of either Mg^2+^ or Mn^2+^ resulted in apparent S_0.5_ values higher than those in the presence of the free activating ions alone. All in all, these results suggest that the inhibitory ion can bind to both the free cation site, leading to a partial enzyme activation (n_H_ and apparent S_0.5_ values reduced), and the Mg-ATP site, lowering the V_max_. Interestingly, the effectiveness of divalent cations, either activatory or inhibitory, seems to be related to their ionic radius. Besides this, the behaviour towards Mg^2+^, Mn^2+^ and Ca^2+^ of *Mtb*PRPPase differed from that of the *B. subtilis* and the human enzymes (both more activated by Mg^2+^ than Mn^2+^) and strongly inhibited by Ca^2+^ [31], [32], [37], thus suggesting a different geometry of the free cation binding site. Figure 10 shows the sequence alignment of the human, *B. subtilis* and *M. tuberculosis* cation binding site, as deduced from the *B. subtilis* structure [35], and obtained using Multalin 5.4.1 [38]. Arg^180^ (*B. subtilis* numbering), in the absence of cation, establishes a hydrogen bonding network with two aspartic acid residues (Asp^174^ and Asp^223^) devoted to the free Mg^2+^ binding, and moves away to a new aspartic acid residue (Asp^133^) in the presence of the ion. In the *Mtb*PRPPase, Arg^180^, which is also conserved in the human enzyme, is substituted by an isoleucine, whereas two arginines are located one and three residues behind, respectively. These structural differences could very likely be the reason for a different free cation site topology, thus accounting for the different ion specificity.

It is known that class I enzymes are allosterically inhibited by purine diphosphate nucleosides [31]–[32]. *Mtb*PRPPase acted as the enzymes of this class (Fig. 7A), with non-competitive inhibition by Mg-ADP, either in the absence or in the presence of free Mg^2+^. Similarly to the *B. subtilis* and *Salmonella typhimurium* enzymes [31], [39], *Mtb*PRPPase was only weakly inhibited by Mg-GDP, distinguishing itself from the mammal enzymes which were more affected by this nucleotide (IC_50_, 10-fold higher) [32]–[33]. On the other hand, *Mtb*PRPPase was more sensitive to inhibition by ADP than *B. subtilis* enzyme (IC_50_, 4-fold lower) [31], to this respect behaving like mammal enzymes [32]–[33]. Interestingly, the concentration of the ADP needed by *Mtb*PRPPase for half-maximal inhibition increased with increasing P_i_ concentration, thus supporting the conclusions of previous studies that indicate the presence of a regulatory site to which both inhibitory ADP and activatory P_i_ could bind [22]. That *Mtb*PRPPase was regulated by ADP in an allosteric manner resulted by the kinetic responses to substrates concentrations at two different concentrations of ADP. In fact (Figure 7A and 7B, Table 5 and 6) V_max_ was the only parameter affected. Therefore, *Mtb*PRPPase underwent the inhibition by ADP fully meeting the uncommon requirements of the *V*-type allosteric enzyme described by Monod *et al.* [36].

In conclusion, the biochemical investigation on PRPPase from *M. tuberculosis* allows us to add a well-characterized member to class I enzymes, and to contribute to the elucidation of the regulatory properties of this complex enzyme involved in nucleotides and in the mycobacterial cell wall biosynthesis. The picture emerging from these studies is that of a “chameleon” enzyme which adopts different conformations in response to a variety of allosteric effectors, either positive or negative, thus finely adapting the synthesis of PRPP to the variable cell demands. The enzyme characterization may represent the starting point for the development of inhibitors for antitubercular drug design, also in the light of the structural differences with respect to the human counterpart, as suggested by the *Mtb*PRPPase three dimensional structure prediction. Our model supports the notion that the different kinetics shown by the mycobacterial and human PRPPase are likely due to peculiar structural traits of the nucleoside triphosphate binding pocket and suggests that the identification of selective ligands can be challenged. In this respect, it is worth mentioning that M. tuberculosis ATP phosphoribosyl transferase (HisG) (the enzyme catalysing a reaction one step downstream PRPPase along the same pathway and also showing a significant degree of sequence identity with the human ortholog), has been successfully approached for the discovery of inhibitors selective toward the *M. tuberculosis* enzyme by exploiting the PRPP binding site in structure based virtual screening [40]. Therefore, although we recognise that the issue of the selectivity of inhibitor action is a major concern in the case of *Mtb*PRPPase, both our extensive biochemical investigation as well as a foreseen more robust structural characterization, may prove to be useful for the design of potent and highly specific inhibitors.

## Supporting Information

Figure S1 **Inhibition of** ***Mtb*** **PRPPase by ADP at different P_i_ concentrations.** Response of *Mtb*PRPPase activity to Mg-ADP different concentrations, in the presence of 5 mM (▵) and 50 mM potassium phosphate (▴). All measurements were performed in 50 mM Tris-HCl pH 8.0, at 2 mM R5P and 1 mM Mg-ATP.
(TIF)

Figure S2 **Characterization of the recombinant** ***Mtb*** **PRPPase after the removal of the His-tag.** *Mtb*PRPPase, after the removal of the hexahistine tag, was kinetically characterized and compared with the kinetic properties of the enzyme provided with His-tag. Closed symbols indicate the enzyme with His-tag attached to its N-terminus, open symbols the enzyme without His-tag. (A) Steady state kinetics of enzyme as a function of R5P at fixed 10 mM concentration of Mg-ATP, in the absence of free divalent cations (•), and in the presence of 5 mM MgCl_2_ (▪); (B) Steady state kinetics of *Mtb*PRPPase as a function of Mg-ATP at fixed 2 mM concentration of R5P, in the absence (**•**) and in the presence (▪) of 5 mM MgCl_2_. (C) Response of activity to CuCl_2_ (▴), CaCl_2_ (▾) and FeCl_2_ (▪) different concentrations, at 2 mM R5P and 5 mM Mg-ATP. (D) Response of activity to Mg-ADP different concentrations (▴), at 2 mM R5P and 1 mM Mg-ATP.
(TIF)

Materials and Methods S1 **Expression and Purification of Recombinant** ***Mtb*** **PRPPase Devoid of His-tag.**
(DOC)
