# Supplementary figures and images for: Mycobacterium tuberculosis Phosphoribosylpyrophosphate Synthetase: Biochemical Features of a Crucial Enzyme for Mycobacterial Cell Wall Biosynthesis
Source: PLoS One. 2010 Nov 15;5(11):e15494. doi: 10.1371/journal.pone.0015494 (PMC2981568; doi:10.1371/journal.pone.0015494)

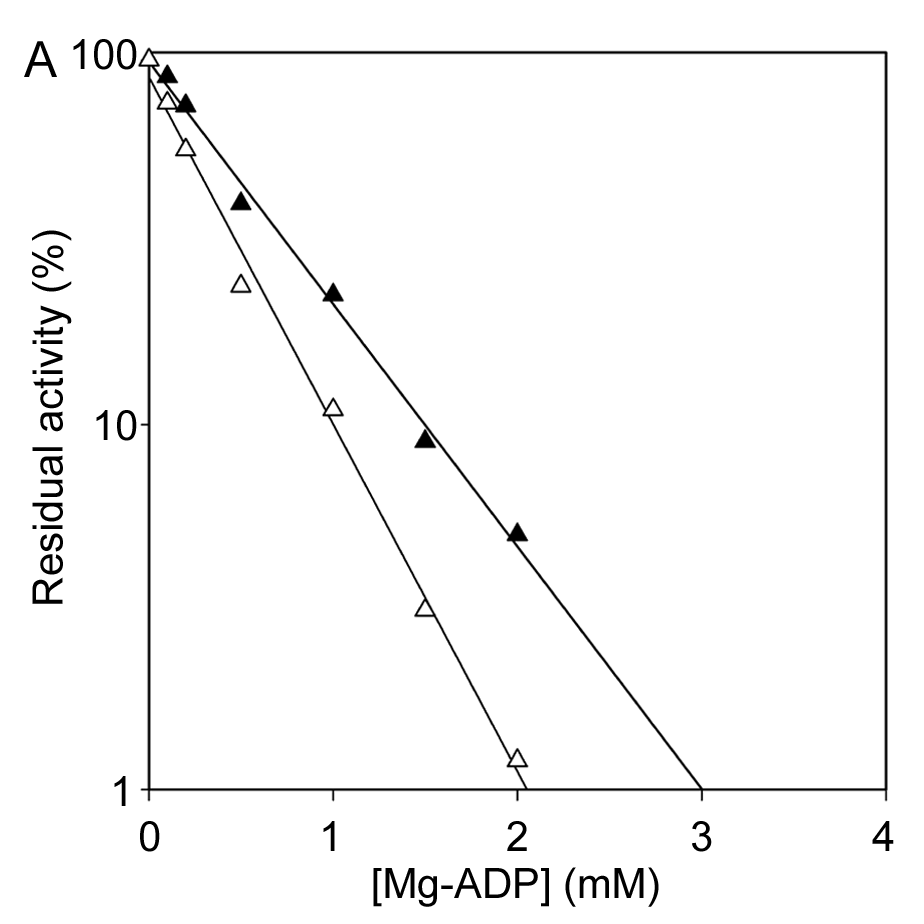

Supplement: Figure S1 — Inhibition of Mtb PRPPase by ADP at different Pi concentrations. Response of MtbPRPPase activity to Mg-ADP different concentrations, in the presence of 5 mM (▵) and 50 mM potassium phosphate (▴). All measurements were performed in 50 mM Tris-HCl pH 8.0, at 2 mM R5P and 1 mM Mg-ATP. (TIF) [file pone.0015494.s001.tif]

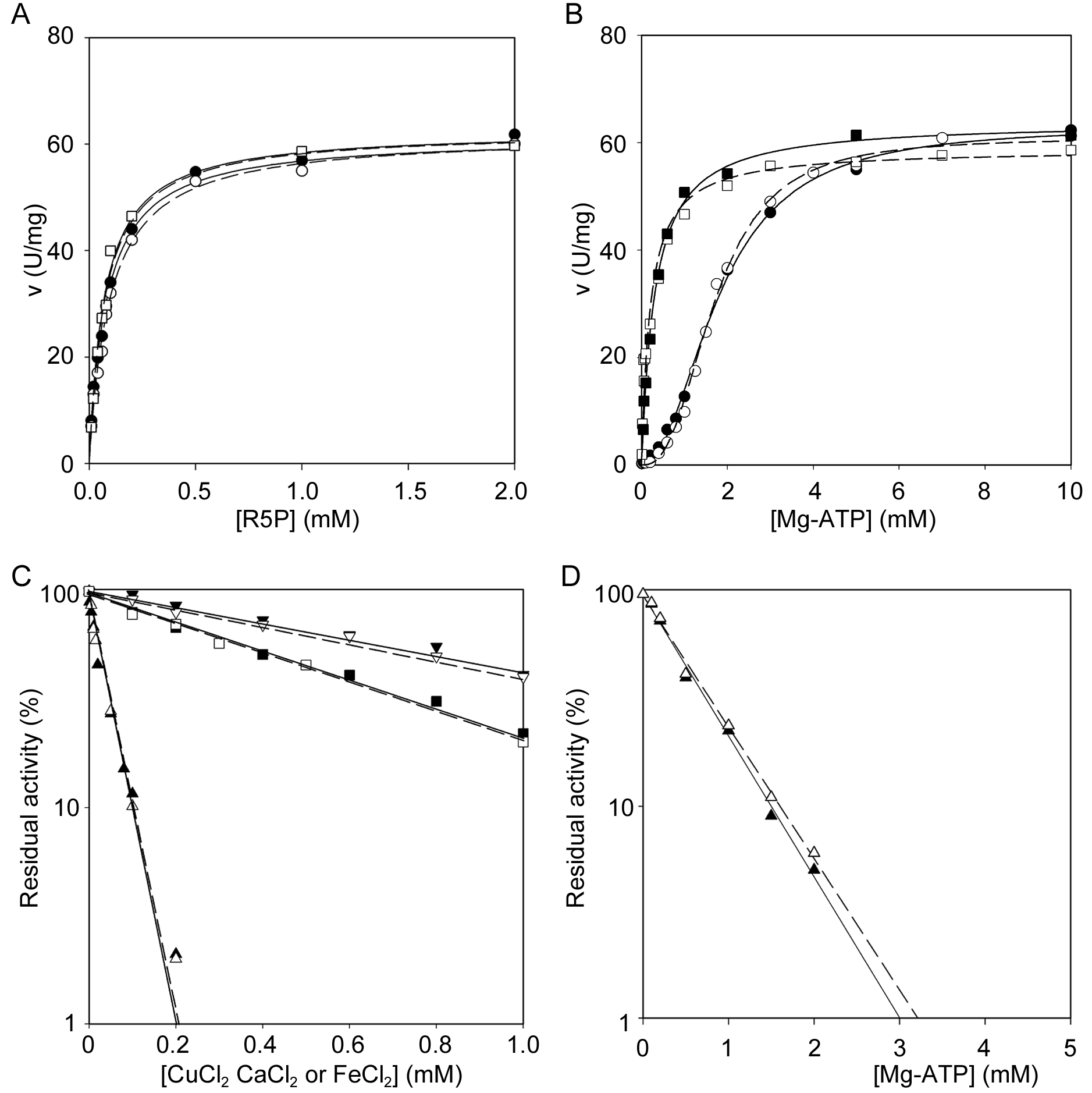

Supplement: Figure S2 — Characterization of the recombinant Mtb PRPPase after the removal of the His-tag. MtbPRPPase, after the removal of the hexahistine tag, was kinetically characterized and compared with the kinetic properties of the enzyme provided with His-tag. Closed symbols indicate the enzyme with His-tag attached to its N-terminus, open symbols the enzyme without His-tag. (A) Steady state kinetics of enzyme as a function of R5P at fixed 10 mM concentration of Mg-ATP, in the absence of free divalent cations (•), and in the presence of 5 mM MgCl2 (▪); (B) Steady state kinetics of MtbPRPPase as a function of Mg-ATP at fixed 2 mM concentration of R5P, in the absence (•) and in the presence (▪) of 5 mM MgCl2. (C) Response of activity to CuCl2 (▴), CaCl2 (▾) and FeCl2 (▪) different concentrations, at 2 mM R5P and 5 mM Mg-ATP. (D) Response of activity to Mg-ADP different concentrations (▴), at 2 mM R5P and 1 mM Mg-ATP. (TIF) [file pone.0015494.s002.tif]
