# Supplementary material for: Mycobacterium tuberculosis Phosphoribosylpyrophosphate Synthetase: Biochemical Features of a Crucial Enzyme for Mycobacterial Cell Wall Biosynthesis
Source: PLoS One. 2010 Nov 15;5(11):e15494. doi: 10.1371/journal.pone.0015494 (PMC2981568; doi:10.1371/journal.pone.0015494)
Supplement: Materials and Methods S1 — Expression and Purification of Recombinant Mtb PRPPase Devoid of His-tag. (DOC) [file pone.0015494.s003.doc]

**Expression and purification of recombinant *Mtb*PRPPase devoid of His-tag**

# To obtain the *Mtb*PRPPase with a cleavable His-tag the *rv1017c* gene was cloned into the pET28-a expression vector, as reported in Material and Methods section, but using the following forward primer: 5’-TTGGATCCCTGGAAGTTCTGTTCCAGGGGCCCTTGAGCCACGACTGG-3’, carrying the sequence encoding the recognition site for the PreScission protease (underlined). The enzyme was expressed and purified with the same procedure described for the uncleavable *Mtb*PRPPase. Furthermore, the protease digestion between the nickel chelate affinity and the size esclusion chromatographies was introduced. As for the proteolytic digestion, active fractions eluted from HisTrap HP column were pooled, dialysed against 50 mM potassium phosphate pH 8.0, 150 mM NaCl, 5 mM EDTA, 1 mM DTT, then incubated for 12 hours at 4°C in the presence of 10 units of PreScission Protease (GE Healthcare). The cleavage was checked by 12% SDS-PAGE and the purification was completed as reported.
